# Gender effects in crowdfunded business loan campaigns

**DOI:** 10.1371/journal.pone.0305601

**Published:** 2024-07-10

**Authors:** Pomme Theunissen, Matteo Millone

**Affiliations:** 1 Department of Data Analytics and Digitalisation, Department of Finance, School of Business and Economics, Maastricht University, Maastricht, The Netherlands; 2 APG Asset Management, Amsterdam, The Netherlands; University of Rome Tor Vergata: Universita degli Studi di Roma Tor Vergata, ITALY

## Abstract

Crowdfunding is a growing source of finance for entrepreneurs. In this paper, we investigate the existence of a gender effect in the time needed to obtain a business loan through crowdfunding. Using data from three Dutch crowdfunding platforms, survival analysis of the time to completion for 934 business loan campaigns shows that female entrepreneurs have a 20% shorter campaign completion time compared to male entrepreneurs, whereas couples do not differ from males. This effect persists across the different platforms. Subsequent analysis shows that female entrepreneurs do not have the disadvantage they face in traditional lending channels when requesting funds through crowdfunding, and that herding behavior by investors benefits female entrepreneurs most.

## Introduction

One of the major challenges for entrepreneurs is access to finance. Obtaining financing is key to scaling up operations, develop new products, and foster innovation [1,2]. Generally speaking, financing start-ups and SME’s is challenging due to the lack of reliable financial track records, and the high levels of asymmetric information which make it difficult for investors to determine the riskiness of an enterprise [1,3,4].

Raising financing is particularly problematic for female entrepreneurs [5,6] In traditional banking, female entrepreneurs have to pay more for credit and face stricter lending conditions than men [7–11]. Interestingly, when requesting a loan for private purposes through a crowdfunding platform, earlier research shows that female borrowers face better [12] or equal [13] *loan conditions* than men. This suggests that females are apparently not at a disadvantage compared to males when it comes to private loan-based crowdfunding. The question remains whether this finding also holds when female entrepreneurs apply for a business loan through crowdfunding.

In a crowdfunding setting, fund seekers request small amounts of money, called pledges, from a large number of investors through the intermediary of a crowdfunding platform [14–16]. Several types of crowdfunding can be distinguished: donation based, reward based, equity based, and loan based (e.g. peer-to peer lending) [17]. The latter is the focus of this study.

We benefit from a unique dataset provided by the Dutch financial advisory platform “Fundwijzer.” Currently, The Netherlands is amongst the largest crowdfunding markets worldwide, with a market size spanning a little over one billion Euro of investments for the year 2022 across the different types of crowdfunding [18]. Our dataset contains campaign pledging information by the minute of three distinct crowdfunding platforms. Through this data, we obtain a detailed overview for 934 business loans granted by Dutch investors to both Dutch entrepreneurs, and entrepreneurs in developing countries. Apart from the distinction between male and female entrepreneurs, the data allow us to distinguish couples (two persons jointly responsible for the loan repayment, either spouses, or (business) partners) who aim to acquire funding for their business.

Our research objectives and contributions are threefold. First, we aim to determine whether business loans requested through crowdfunding show a gender effect when it comes to the time needed to obtain the desired funding and whether this effect is mitigated when a couple applies for the loan. Numerous studies have established that females are successful when seeking funds via crowdfunding [12,19–23]. Yet, to the best of our knowledge, none of these studies has investigated how gender may impact the speed of funding. Time is crucial for entrepreneurs, for example in order to acquire financial resources [24,25], gain market share [26], or enter new markets [27].

Second, we aim to investigate whether a gender effect in the time to completion arises from investors’ herding behavior. In face of uncertainty, when deciding whether or not to provide funding to an entrepreneur, investors have an incentive to interpret the behavior of other investors and have a greater tendency to mimic the investment decisions of peers [28]. This well-established effect in crowdfunding campaigns is called *herding*, where investors show a tendency to follow the pledging behavior of others in their decision to allocate funds to specific crowdfunding campaigns [16,28,29]. Whether this herding behavior may put female entrepreneurs at an advantage or disadvantage compared to their male counterparts is a question this study aims to answer.

Third, we answer the call from Barasinska and Schäfer [13] to conduct a comparative analysis among different platforms, and examine whether any gender related prejudicial behavior towards entrepreneurs is platform dependent. Crowdfunding platforms mitigate the information asymmetry between investors and founders more efficiently than investors do on their own [17]. Given their role as intermediaries, having data from three different platforms with heterogenous characteristics enables us to determine whether a possible gender effect in crowdfunding campaigns could be platform dependent.

We find that crowdfunding loans initiated by female entrepreneurs have a 20% shorter campaign completion time compared to loans initiated by male entrepreneurs. The loan completion hazards of entrepreneur couples do not significantly differ from those of male entrepreneurs. We show that the gender effect is persistent across different crowdfunding platforms, and that female entrepreneurs benefit most from investors’ herding behavior.

The remaining of the paper is organized as follows: the Related literature section provides an overview of the relevant literature on loan-based crowdfunding, gender effects in crowdfunding, and the dynamics of crowdfunding (loan) markets. The Data section describes the data, the characteristics and the loan process of the three crowdfunding platforms. The following section explains the identification strategy in our Cox proportional hazard regression. The Results section shows the results of the survival analysis and additional robustness analyses. The final section concludes.

## Related literature

### Loan-based crowdfunding

Crowdfunding has emerged as an alternative to bank lending for entrepreneurs who seek funding for their business [1,14,30]. Loan-based crowdfunding, or peer-to-peer lending, can be distinguished from traditional lending channels in several ways. First, in loan-based crowdfunding, borrowers gather small amounts of money, pledges, from a large number of lenders [15,16]. Second, lenders and borrowers in peer-to-peer lending markets are brought together directly, most often through an online crowdfunding platform [14,30]. Finally, although lenders have information on the amount pledged by other investors to a particular campaign, they rarely have reliable information on the default risk of borrowers [16,31].

When it comes to assessing a borrower’s credit profile, crowdfunding offers several advantages over traditional loan markets that may be favorable to females. First, the degree to which borrowers provide personal information may be beyond what is asked by traditional banking institutions, where the legal framework may prohibit them from asking certain types of questions [12]. Second, it is the “wisdom of the crowd” that assesses the borrowers’ credit worthiness, as opposed to the loan officer(s) or bank clerk(s), where it is a single, or small number of individuals, who take the decision to grant the requested loan [14,15,32–34]. Previous studies have established that bank loan officer’s taste may drive gender-based discrimination in credit markets [8,35], and that sex prejudices may guide their decisions when considering loan applications by female entrepreneurs, because females are perceived, or feel to be perceived, as less entrepreneurial than men [36–38]. Third, the amount of soft information provided to the investors in a crowdfunding setting can be interpreted as a signal of trustworthiness and as such makes it easier to evaluate the creditworthiness of the borrower [31,32,39]. The availability of soft information also reduces uncertainty for banks [40]. However, obtaining such soft information is a time consuming and lengthy process, which needs to be channeled through the different layers of decision making present at the bank, before it is incorporated in lending decisions [41].

The information provided on the crowdfunding platforms guides the investors in their funding decisions and ultimately determines whether the campaigns will be fully funded or not. Successful crowdfunding campaigns share several characteristics: borrowers who offer higher rates of interest [13] or show favorable credit profiles [15] have an increased probability of reaching their target. On the other hand, fund seekers that request relatively larger target amounts face a decreased probability of success [13]. Further success determinants include the prominence of personal networks [30,42,43], frequent communication with the crowd [30], geographical and cultural proximity [44], and campaign communication and linguistics [45–49].

Several authors have highlighted the importance of providing visual information, such as pictures or videos of the entrepreneur, on the campaign webpage [15,50]. In uncertain investment contexts characterized by high levels of asymmetric information, investors might use mental short cuts, called heuristics [51], and other cues readily available in the environment to assess the firm’s quality [52]. Visual representations of the entrepreneur provide additional information on the fund seeker beyond purely facial information [53]. Pictures, or videos, allow for self- presentation [50], and physical features can be used as replacement for unobserved attributes that correlate with positive behavioral characteristics, such as competence or trustworthiness [54]. For example, the choice of clothing, pose and expressions also deliver behavioral cues to investors [53]. In peer-to-peer loan markets, having a trustworthy appearance affects the chances of obtaining the loan and the level of the corresponding loan rate [55], whereas investors discriminate against borrowers who are elderly or overweight [12]. Attractive borrowers benefit from the “beauty premium” [56]; they are more likely to obtain a loan, pay similar loan rates than average looking borrowers, whereas they default more often [57]. In the setting of initial coin offerings (ICO’s), confident looking management teams raise more funds [50], and attractive CEO’s are found to have a positive relationship with firm valuation [54].

Some studies show that attractiveness matters most for women when raising funds. Female, blond, solicitors are found to elicit greater contributions in door-to-door fund raising [58], and attractive female recipients of charitable crowdfunding campaigns receive more donations from male donors than unattractive females [59]. Other studies conclude that attractiveness matters for male entrepreneurs, and fail to find conclusive evidence for female entrepreneurs in their samples [60–62]. Even more importantly, it is the absence of visual information that appears to trigger most negative responses from investors, as this absence makes it less likely to get funded [12].

Crowdfunding platforms themselves also play a crucial role in mitigating the information asymmetry between investors and fund seekers [17,39]. Platforms are for profit organizations and mainly base their revenues on the fees paid by the fund seekers [63–65], and therefore need to act as trusted intermediaries [64]. This can be related to the renowned “lemons problem”: a platform offering mostly low quality campaigns will be shunned by high quality fund seekers out of fear of not being recognized as high quality and thus failing to obtain their requested loan amount [64,66]. As such, platforms have the incentive to maintain high standards when matching investors and fund seekers, in order to safeguard their reputation. This can be done by thoroughly screening the fund seekers and their campaigns before publication on the platform website, by increasing overall transparency of the funding process, by implementing a reliable borrower credit risk management system, and by promoting a track record of successful previous campaigns [31,64,65].

All things considered; loan-based crowdfunding thus offers several advantages for female entrepreneurs in comparison to loan applications in traditional lending markets. Given the problem of asymmetric information faced by investors when making financial decisions, the setting of the crowdfunding campaigns in which entrepreneurs can disclose as much (soft) information as they want, combined with the incentives of the platform to thoroughly screen the loan campaigns they offer, female entrepreneurs are the ones that likely benefit most from this increased informational transparency towards potential investors.

### Gender effects in crowdfunding

When investigating the gender effects in our study, we take the perspective of role congruity theory to explain why our findings may seem at odds with earlier research findings on gender in entrepreneurial finance. Role congruity theory contends that if individuals hold a stereotype about a social group that is incongruent with the attributes that are believed to be required to attain success in certain social roles, this inconsistency will lower the evaluation of the group member that occupies that role [67]. In other words, if prospective investors hold certain stereotypes about women that are at odds with the attributes that are believed to be necessary to be a successful entrepreneur, this incongruence will lower the evaluation of women as entrepreneurs, and hence make it more difficult for them to raise financing. A strict interpretation of this incongruence is important here: being feminine is not in itself the reason why females are less successful at raising financing, it is the perceived antonymous element in *female* entrepreneurship that is at the root core.

That nuance matters, because Heilman et al. [68] have shown that a simple dichotomy of gender may be an oversimplification. Rather, core feminine and masculine characteristics may offer the explanation as to why women are more successful in crowdfunding settings compared to males [12,19,20–23]. Signals about these characteristics can thereby positively affect success in raising financing, as long as those signals are congruent with the stereotypical gender role [46,47,69]. Crowdfunding platforms, by their very nature, are built in part to allow entrepreneurs to give credible signals about the characteristics of their project … and themselves. Cowden et al. [19] analyze the impact of agreeableness and humility, two feminine characteristics, and assertiveness and emotional stability, two masculine characteristics, on the crowdfunding success of male and female entrepreneurs. Whereas traditional fund providers have a bias against entrepreneurs with stereotypical feminine traits [70], lenders on crowdfunding platforms align with gender role congruity, and tend to invest in females that are perceived to have feminine characteristics, and invest in males that are perceived to have masculine characteristics [19,71]. Hence, gender stereotypes play out differently in crowdfunding than in traditional financing, where it is the alignment of gender roles that is positively associated with crowdfunding success [19].

Various studies show that female entrepreneurs enjoy higher rates of successful campaign completion than male entrepreneurs in a pre-sale crowdfunding setting based on data from the crowdfunding platform *Kickstarter*. This finding is explained in several ways: first, male overconfidence causes male entrepreneurs to set overly optimistic targets for their campaigns, whereas females set more realistic goals which are thus more likely to be achieved [22]. Second, investors perceive females to be more trustworthy than their male counterparts [23]. Third, Gafni et al. [20] show that crowdfunding projects led by females have higher success rates than male-led projects, and that this success is not driven by the mere fact that females request smaller amounts of funds, nor by the type of the project, such as for example art, comics, dance, etc… But rather, this success may stem from a supply side effect: female investors are more likely to finance female-led projects on crowdfunding platforms. In a similar vein, Greenberg & Mollick [21] explain the success of female founders as being driven by activist choice homophily–the feeling of belonging to a social group in which support helps to overcome common barriers: women are inclined to help other women in breaking barriers, primarily in industry categories in which females are underrepresented.

In private peer-to-peer loan settings, not related to a business but for private purposes, the findings on female borrowers are rather mixed. Pope and Sydnor [12] find that in the U.S. women have a higher likelihood of obtaining funding and pay lower interest rates than men, whereas Barasinska and Schäfer [13] do not find a gender effect in borrowing success on the German peer-to-peer lending platform *Smava*. The latter argue that this difference in findings may be due to the difference in the existence of a bank-based loan system in Germany compared to the US market-based system. Barasinska and Schäfer [13] also find that females request on average smaller loan amounts than men; where a larger amount of funds relates negatively to campaign success [30,72].

In contrast, in equity-based crowdfunding settings, research findings tend to reveal the presence of a gender gap in financing at the detriment of females. Geiger & Oranburg [73] show that equity crowdfunding campaigns initiated by females attract significantly less funding. Similarly, Prokop & Wang [74] find that when securing seasoned rounds of financing, females are less successful compared to their male counterparts, attracting less capital as well. One possible explanation for the presence of such a gender gap is that investors respond differently to signals of media coverage and management experience coming from the entrepreneurs. If the signal from the female entrepreneur is contrary to her stereotypical gender role, equity investors are less likely to pledge their funds [69]. This latter argument brings us back to role congruity theory, and the importance of aligning gender roles when applying for funds via crowdfunding.

### Herding

As mentioned in the introduction, the *herding* effect is well established within the crowdfunding literature [16,28,29,75,76]. Herding behavior is defined as decision makers mimicking the behavior of other decision makers instead of using their own private information [77]. This behavior is rational because other individuals may have information that is unavailable to the decision maker. When facing investment decisions with a high degree of uncertainty and asymmetric information, such as in peer-to-peer loans, investors observe lending decisions of peers to infer the creditworthiness of borrowers [16] and have a higher tendency to herd [76].

Crowdfunding platforms provide information on the amount of funds already pledged for each campaign as an indication of how close the campaign is to reaching the target amount. On most platforms, the loan will only materialize if this target amount is attained, this is the provision point mechanism [75]. If the target is not reached, investors face opportunity costs (the invested amount of money is not pledged elsewhere) and additional search cost: looking for another interesting investment opportunity [16,28]. Investors thus have an incentive to invest in campaigns that are close to attaining their target amounts.

Crowdfunding campaigns that have attracted a large number of pledges are more likely to attract even more pledges [28], both in terms of the number of investors, and in terms of the amount of funds pledged [78]. Investors thus take previous funding as a positive signal for their own investment decision [29]. Pledges done in the early phases of the campaign signal that the borrower can be perceived as trustworthy [28], and are closely associated with a successful campaign outcome [78,79].

In an equity crowdfunding setting, Mohammadi & Shafi [80] analyze possible gender effects on the investor side. Drawing on the risk aversion literature, they show that female investors are less likely to invest in young firms, in high technology firms, and in firms that offer a higher share of their equity—therewith giving up a higher share of ownership. Interestingly, the authors also show that female investors are more likely to invest in firms with a higher proportion of male investors, therewith providing evidence of a supply side gender-related herding effect, with female investors mimicking the investment behavior of male investors rather than female investors.

Most studies see the presence of herding as a positive effect because herding associates with increased campaign success. However, herding may also introduce an inefficiency in the dynamics of the fund pledging process [65]. Campaigns that receive funds quickly in early stages are more likely to succeed than other campaigns, but may actually not be the ones that are the most beneficial from an investor perspective. Moreover, the early pledges can be engineered from social networks purposefully, in order to increase the probability of success [42,81].

Given the asymmetric information and the high level of uncertainty involved in peer-to-peer loans, investors in loan-based crowdfunding are likely to take the behavior of their fellow investors into consideration when making their investment decision. Increasing the level of available information may therefore reduce investor uncertainty about borrowers, and may weaken the need to herd [16,82].

### Speed of campaign funding

Only a few studies analyze the speed of funding completion in crowdfunding campaigns. In certain commercial settings, the time necessary to obtain funds matters greatly, for example when the products brought to market by the entrepreneur are perishable [83]. Moreover, obtaining funds swiftly is relevant to all entrepreneurs, as financing is a key driver of business growth and development [84]. The longer it takes to raise funds, the longer other crucial tasks like technology advancement, R&D, and human capital investment have to wait [85].

Several factors play a role in determining the speed of funding. In a large scale study of over 6,000 loan applications of developing country entrepreneurs, Allison et al. [86] show that the narratives used in the loan applications play a key role. Funding speed is slower when entrepreneurs have more variety in their rhetoric and when the rhetoric used is associated with accomplishments. Funding speed is found to be greater when entrepreneurs employ rhetoric associated with blame and present concern. According to dos Santos Felipe et al. [87] who conduct a study with data from a Brazilian crowdfunding platform, the campaigns that attain their target most rapidly are the ones located in cities with greater income inequality.

Ahlers et al. [88] investigate the speed of campaign completion determinants in an equity based crowdfunding setting. The authors show that the availability of information related to internal governance and the level of retained equity, in sum, factors that decrease information asymmetry, not only increases the probability of success for equity crowdfunding campaigns, but also increases the speed of funding.

Our finding that female entrepreneurs benefit more from herding than their male counterparts is likely to be a combined effect of (a drop in) incongruence and asymmetric information. First, as we have already explained, because crowdfunding platforms allow for a greater amount of (soft) information compared to traditional lending markets, female entrepreneurs can assure that their gender role aligns with their feminine characteristics in the eye of investors, lowering incongruence. Second, as business loans offered via crowdfunding platforms are characterized by high levels of asymmetric information, investors will focus on the campaigns that allow the greatest levels of information inference, increasing the speed at which funds are pledged. Combined with the first effect, the resulting herding by investors benefits females most. This effect may be strengthened even further if more high-quality female borrowers are rejected in traditional loan markets because of the presence of a gender bias. If these females search for alternatives and flow to crowdfunding markets instead, these females are then on average more creditworthy than their male counterparts, further stimulating investors’ tendency to herd when financing these females in crowdfunding markets.

## Data

### Platforms

Our dataset is obtained through the Dutch financial advisory platform “*Fundwijzer*,” and contains pledging information for three Dutch crowdfunding platforms. For the purpose of this study, we anonymize these platforms and identify them by CFP1, CFP2 and CFP3, respectively. The available information allows us to obtain a detailed overview on the temporal evolution of the pledges for 934 crowdfunding loan campaigns. These campaigns took place between June 2014 and July 2016, and all successfully reached the required loan amount. The data consists of the name of the project, the name of the crowdfunding platform and the related URL, the target loan amount requested by the entrepreneur, the total amount pledged at each scraping moment (expressed in date, hour, minutes and seconds), the start time of the campaign, and the end time of the campaign. With the provided URL, we supplement the data manually with the information on the gender of the entrepreneur, the monthly interest rate and the maturity of the loan, and whether entrepreneurs provided a picture or a video representing him-, her- or themselves. Finally, we followed Calic & Mosakowski [89] to determine whether or not the project can be classified as “sustainable.”

CFP1 is a crowdfunding platform based in the Netherlands that enables fund pledgers to invest in entrepreneurs and sustainable initiatives in developing countries. The platform focuses on social impact investing through the provision of meso-credits.

When investing in a loan through CFP1, investors buy bonds with semi-annual coupon payments, and a nominal value of €50 each. If the bond denomination is in foreign currency, the purchase price is paid in Euro based on the Euro counter value. The interest payment to be received is calculated on the outstanding amount of the relevant loan. The loan maturity may vary between 6 and 48 months, and the loan rates are set between 3% and 6%.

The local partners of the crowdfunding platform within the different countries assess the credit worthiness of the projects; the platform itself thus only serves as instrument between the entrepreneurs and the investors. Campaigns are closed upon reaching the requested investment amount and stay open for subscription at most 60 days. The platform holds an “all-or-nothing” approach, and projects cannot be overfunded beyond the initial requested loan amount. Although CFP1 does not provide a credit rating for the campaigns, the platform provides the financial statements and detailed information regarding the investment purpose of the loan. The average yield spread charged by CFP1 on the loans is 3.1%. Investors do not pay any fees to participate in the crowdfunding campaign, only if they pay with credit card will they be charged 1.5% on the amount invested, plus a €0.20 fee.

CFP2 is one of the leading crowdfunding platforms of the Netherlands, and is accessible to both Dutch entrepreneurs and private individuals to request a loan. The platform does not specify what the share of delayed repayments is; neither does it clearly specify the share of unsuccessful campaigns.

Entrepreneurs set the loan conditions in terms of the type of loan (annuity, non-amortizing loan, or interest-only loan), its maturity and the loan rate, with the advice of CFP2. The maturity of the loan can vary from several months up to a maximum of 10 years and loan rates may vary between 4% and 12%. CFP2 screens the entrepreneurs for solvability and establishes a credit rating based on several measures such as their credit registry score BKR (“*bureau krediet registratie”*), the credit safe score and the Graydon Probability of default. Campaigns are closed upon reaching the requested investment amount and stay open for subscription for a maximum of 60 days. The platform uses an “all-or-nothing” approach: if the project does not reach the requested loan amount, investors receive their pledges back.

Investors make incremental promises of payment with a minimum of €100 for the project of their choice, with the payment required only when the project reaches the target loan amount desired by the entrepreneur. Investors pay a success fee of minimum 0,4% annually on the loan amount they invested. CFP2 charges publication costs ranging between €125 and €499 for each campaign that is offered on the platform. Additionally, when the campaign is successful the platform will charge the entrepreneur a onetime success fee of at least 1.5% on the loan amount obtained, depending on the loan type and the total amount of funds obtained.

CFP3 is a crowdfunding platform solely dedicated to Dutch entrepreneurs and SMEs. The platform conducts extensive due diligence when it comes to the entrepreneurs, including company visits, accounting checks, and provides “CFP3-coaches” who help the entrepreneurs determine their funding needs and loan conditions. Loan maturity ranges from 6 months up to a maximum of 10 years. Loan rates vary between 5% and 9% based on the credit score awarded by the platform, the Dun & Bradstreet risk score and the CFP3-credit score, to signal the entrepreneurs’ risk level to the investors. Investors provide incremental pledges of €500 via a CFP3 investment account, and pay a monthly administration fee of 0.1% on the remaining loan amount. The platform indicates a success rate of 99.5% for its campaigns, and has written-off 3.71% of the provided loans.

Campaigns are closed upon reaching the requested investment amount, and stay open for subscription for a maximum of 31 days. The platform holds an “all-or-nothing” approach with no overfunding possibility beyond the initial requested loan amount. If the campaign is not completed after 31 days, the platform will decide whether or not to extend this period on a case-by-case basis. CFP3 charges credit evaluation costs of €350, a publication fee of €850, a monthly administration fee of 0.07%, and a success fee of 1.5% (with a minimum of €1,500) on the obtained loan amount.

The platform descriptions show that the three platforms differ substantially from each other on certain aspects. CFP1 provides loans to developing country entrepreneurs, CFP2 provides loans to both private individuals and entrepreneurs, and CFP3 only provides loans to Dutch entrepreneurs. The maximum campaign time for CFP1 and CFP2 is 60 days, and only 31 days for CFP3. Minimum pledges for CFP1 are €50, for CFP2 €100, and €500 for CFP3. In terms of fees and costs, all three platforms differ substantially as well. Table A in S1 Appendix provides a detailed comparison of the platforms.

Moreover, the three platforms do share a similar “all-or-nothing” approach regarding the amount of funding obtained. This entails that entrepreneurs will only receive the requested funds if the target loan amount is reached. Cumming et al. [90] find that investors perceive such a choice of “all-or-nothing” funding model—as opposed to the “keep-it-all” model in reward-based crowdfunding as a signal of commitment by the entrepreneurs.

### Summary statistics

Table 1 provides summary statistics for the three crowdfunding platforms. CFP3 statistically differs from CFP1 on all variables. More specifically, the average loan value of the loans requested trough the CFP3 platform is much higher than the loans requested on the CFP1 platform, € 194,000 compared to € 9,384. The average interest rate is higher, and the maturity of the loans longer. CFP1 has a significantly higher share of female entrepreneurs.

**Table 1:** Summary statistics by crowdfunding platform.

	CFP1					CFP2				CFP3			
	mean	median		min	max	mean	median	min	max	mean	median	min	max
Loan value (€)	9,385	5,200		1,800	250,000	130,769	110,000	15,000	600,000	194,160	190,000	50,000	500,000
Interest rate (%)	3.43	3		3	4	7.75	8	4	9	7.5	7,5	6	9
Maturity (months)	24	24		6	48	53	60	15	84	51	54	8	120
Sustainable project	2%					0				6.25%			
Female entrepreneur	52%					13.7%				11,7%			
Couples entrepreneur	5.6%					16.6%				15.6%			
Time to complete (in minutes)	5,928		1,990	30	73,441	6,847	594	0.05	79,367	3,740	200	0.05	37,783
N	705					101				128			

Note: All variables statistically significantly differ between CFP1 and CFP3.

CFP2 does not statistically differ from CFP3 on loan maturity and gender of the borrower.

CFP2 does not statistically differ from CFP1 on time to completion.

The maximum campaign time of 60 days corresponds to 86,400 minutes.

The loan amount requested by entrepreneurs on the CFP3 platform is also significantly higher than the amount requested on the CFP2 platform. The maturity of the loans, and the gender of the entrepreneur does not significantly differ among these platforms. As expected, time to completion is not significantly different between CFP1 and CFP2, as both cap their campaigns at 60 days. The minimum campaign time of 0.05 minutes may seem surprising at first glance, but this is due to pre-announcements by the platforms. Before launching a new campaign, the platforms send e-mails to subscribed potential investors containing relevant loan and business information. This is comparable to rock star concerts that are sold out as soon as the ticket sale goes live.

Table 2 gives the summary statistics for the complete sample and the three entrepreneur types: male, female and couples. In line with the literature, female entrepreneurs request lower average loan amounts; pay lower average interest rates; and have shorter average loan maturities than their male, or couples, counterparts. The requested loan amount is not significantly different between male and couples entrepreneurs; however, couples do pay significantly higher interest rates, and have longer loan maturity than males. Time to completion does not significantly differ between any of the three groups.

**Table 2:** Summary statistics for the complete sample of crowdfunding campaigns.

	Full sample			Male			Female			Couples		
	mean	min	max	mean	min	max	mean	min	max	mean	min	max
Loan value (€)	47,833	1,800	600,000	69,843	1,800	600,000	14,718	1,800	400,000	88,516	1,800	425,000
Interest rate (%)	4.45	3	9	4.9	3	9	3.7	3	8.5	5.57	3	9
Maturity (months)	31	6	120	33	6	120	26	6	60	42	6	60
Sustainable project	2.2%			3%			1.2%			2.5%		
Female entrepreneur	42%											
Couples entrepreneur	19%											
Time to complete (in minutes)	5,728	0.05	79,367	6,184	0.05	79,367	5,376	0.05	50,362	4,843	23	51,734
N	934			458			399			77		

Note: All variables statistically significantly differ between male and female borrowers except time to completion.

Males and couples do not statistically differ on the time to completion, the loan amount and the percentage of sustainable projects.

Females and couples do not statistically differ on the time to completion and the percentage of sustainable projects.

## Identification strategy

### Cox proportional hazards model

We use survival analysis to evaluate the time necessary until the completion of the crowdfunding campaign, measured by the speed at which the target amount of funding is reached [86,88]. Our unit of measure is time, which we report in minutes for the ease of interpretation. In order to measure the difference in speed between the campaign completion time of male, female and couples entrepreneurs, we use a Cox proportional hazards model [91]. This is a semiparametric regression model that makes no assumptions about the shape of the baseline hazard function, while allowing to condition the time to completion on a number of control variables [92].

We take the hazard to be the completion of the campaign; the hazard rate for the *j*th crowdfunding campaign in the data can then be specified as:

$$h_{j}(t|{gender}_{j})=h_{0}(t)\exp{(\beta }_{1}{female}_{j}+\beta_{2}{couples}_{j}+\beta_{3}{herding}_{j}+\beta_{4}X_{j})$$ (1)

Where: t = the campaign time measured in date, hours, minutes and seconds notation: time to completion of the campaign–start time of the campaign. j = crowdfunding campaign.

The coefficients *β*_1_ and *β*_2_ are our coefficients of interest: they estimate to what extent the hazard rate of female entrepreneurs, or couples entrepreneurs, exceeds the rate of male entrepreneurs. The coefficient *β*_3_ estimates to what extend the herding effect impacts the speed of campaign completion. *X_j_* is a vector of campaign specific control variables detailed below.

### Cox proportional hazard model with shared frailty

In entrepreneurship studies, unobserved heterogeneity may be present for example at the level of the country, the market, the industry, or the venture in which the entrepreneur is active [93]. This can be problematic when applying time-to-event models, where so-called spurious duration dependency may lead to biases in the estimation results [93]. In our study, such heterogeneity may be present at the platform level, where one, or several platforms, may simply be more effective at gathering funds than others. Indeed, for the covariates in our analysis, variance between platforms tends to be much higher than variance within platforms. Therefore, unobserved, latent heterogeneity from different platforms may mean that the proportionality assumption in the ‘traditional’ Cox model is violated. As a result, the single hazard resulting from that Cox proportional hazards model would then obfuscate important cross-platform differences. Important for the purpose of our analysis, is especially to find out whether accounting for those differences affects the impact female entrepreneurs have on the hazard. One solution to deal with such unobserved heterogeneity is to apply a shared frailty approach to our Cox proportional hazards model [91,94]. Stated simply, this procedure is analog to a random effects model in regression analysis, where the frailties are common (hence “shared”) among the groups of individuals and are randomly distributed across the groups [91].

When using a Cox model specification, a frailty is a latent random effect that enters multiplicatively on the hazard function [91], and takes the following form:

$$h_{ij}(t|{gender}_{j})=h_{0}(t)\alpha_{i}\exp{(\beta }_{1}{female}_{j}+\beta_{2}{couples}_{j}+\beta_{3}{herding}_{j}+\beta_{4}X_{j})$$ (2)

Where: t = the campaign time measured in date, hours, minutes and seconds notation: time to completion of the campaign–start time of the campaign.

j = crowdfunding campaign.

i = crowdfunding platform.

*α* = the group level frailty.

### Selected variables and model building

Our main variables of interest identify the gender of the entrepreneur. More specifically, whether the borrower is a female entrepreneur, or a couple entrepreneur, measured against the reference category male entrepreneur. Our second variable of interest is the percentage of the loan amount reached in the previous pledging round of the campaign, and serves as a proxy measure for the herding effect.

In order to conduct a comparative analysis amongst the three crowdfunding platforms, we include a categorical variable capturing on which of the three crowdfunding platforms the campaign is offered. Furthermore, to control for possible platform effects we add interaction terms between the gender of the entrepreneur and the platform variable to assess whether the gender effect is possibly platform dependent.

Finally, we include several variables that are found to matter when measuring crowdfunding campaign success. We include the natural logarithm of the requested loan amount in Euros, the loan maturity expressed in months, and the interest rate to be paid on the loan. Additionally, we add a dummy variable that takes the value 1 if the campaign information provided on the website contains a picture (or video) of the entrepreneur, and another dummy variable that takes the value 1 if the project is considered sustainable.

## Results

### Survival analysis

**Fig 1:**
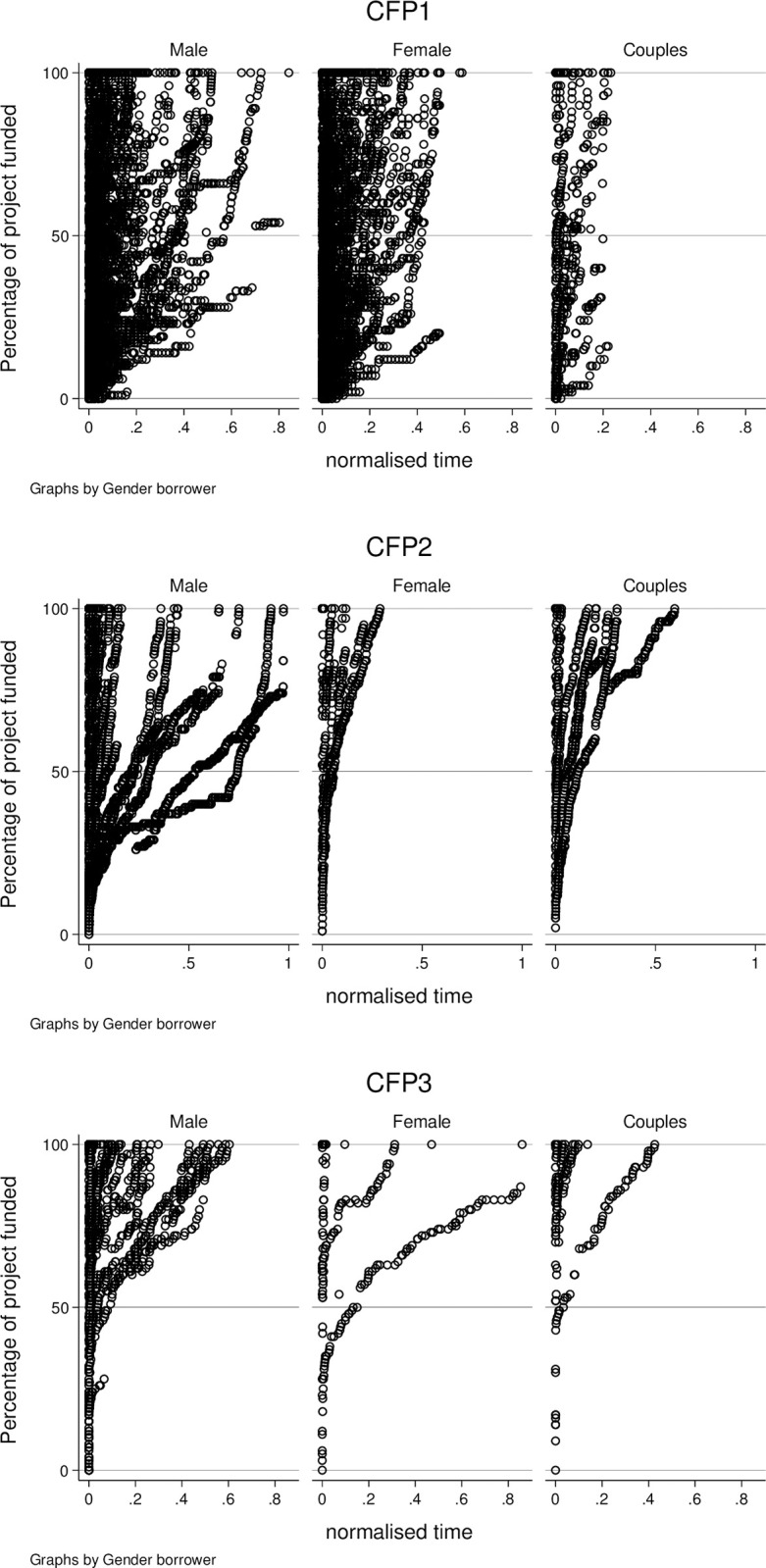
Patterns of pledges by gender for each crowdfunding platform. The plots show the evolution of the pledges as percentage of the requested loan amount obtained over the normalized campaign time, by gender, for each platform.

Fig 1 shows the pattern of pledges by gender for the three crowdfunding platforms. The vertical axis shows the percentage of the requested loan amount that is funded; the horizontal axis shows the normalized campaign time relative to the maximum time allowed on each platform. As stated, this is 60 days for both CFP1 and CFP2, and 31 days for CFP3. Each dot represents a pledge by an investor. At first glance, for both the platforms CFP1 and CFP2, campaigns initiated by female entrepreneurs seem to reach the required loan amount much faster than campaigns initiated by male entrepreneurs. However, for the campaigns displayed on the CFP3 platform, the difference between males and females is less pronounced.

Table 3 shows the results of our Cox regressions. We report the hazard ratios, exp(*β*), meaning that a coefficient greater than 1 indicates an increase in the hazard, i.e., attaining the requested loan amount faster, for each unit change in the predictor variable. Standard errors are clustered at the crowdfunding campaign level.

**Table 3:** Time to crowdfunding loan campaign completion, Cox proportional hazard rate model.

	(1)	(2)	(3)	(4)	(5)	(6)
	model 1	model 2	model 3	model 4	model 5	model 6
LN loan amount	2.1939***	1.2580***	2.2980***	1.3157***	2.2988***	1.3146***
	(0.0000)	(0.0000)	(0.0000)	(0.0000)	(0.0000)	(0.0000)
Loan maturity	1.0092	1.0147***	1.0095	1.0135***	1.0089	1.0131***
	(0.1007)	(0.0002)	(0.1319)	(0.0007)	(0.1651)	(0.0011)
Loan interest rate	0.5028***	0.5887***	0.5921***	0.6760***	0.6029***	0.6820***
	(0.0000)	(0.0000)	(0.0000)	(0.0000)	(0.0000)	(0.0000)
Female entrepreneur (ref: male)	1.4584***	1.2291**	1.4420***	1.1947**	1.4936***	1.1884*
	(0.0005)	(0.0134)	(0.0011)	(0.0352)	(0.0016)	(0.0625)
Couples entrepreneur (ref: male)	1.2138	1.1369	1.2035	1.1355	1.5124**	1.3053
	(0.2019)	(0.3903)	(0.2417)	(0.4001)	(0.0221)	(0.1710)
Picture entrepreneur	1.1663	1.0023	1.4222***	1.2837**	1.4370***	1.2982***
	(0.1237)	(0.9774)	(0.0065)	(0.0119)	(0.0049)	(0.0069)
Sustainable project	0.6163**	0.6283***	0.6304**	0.6640**	0.6176**	0.6588**
	(0.0215)	(0.0057)	(0.0492)	(0.0302)	(0.0407)	(0.0305)
CFP2			0.6374	0.8547	0.6668	0.8872
			(0.3236)	(0.6155)	(0.3899)	(0.7018)
CFP3			0.3203**	0.3491***	0.3287**	0.3345***
			(0.0149)	(0.0012)	(0.0194)	(0.0013)
Female*CFP2					0.9243	0.8874
					(0.8225)	(0.6550)
Female*CFP3					0.8338	1.4849
					(0.7086)	(0.2347)
Couples*CFP2					0.5848	0.7268
					(0.2447)	(0.4701)
Couples*CFP3					0.6773	0.7856
					(0.2313)	(0.4519)
Herding proxy		1.0472***		1.0472***		1.0473***
(Percentage obtained t-1)		(0.0000)		(0.0000)		(0.0000)
Observations	14,365	14,365	14,365	14,365	14,365	14,365
Wald chi^2	272.2	1301	266.9	1287	282.5	1293
No. of campaigns	934	934	934	934	934	934
Funded end of sample	930	930	930	930	930	930

Robust p-values are reported in parentheses.

***, **, and * indicate statistical significance at the 1%, 5% and 10% level, respectively.

In our baseline specification model 1, we exclude our proxy variable for the herding effect. Compared to male entrepreneurs, we find a positive and significant increase of 46% in the hazard of campaign completion when the entrepreneur is female. In line with the findings of Greenberg & Mollick [21] who report that male entrepreneur teams and gender mixed teams do not significantly differ in their probability to raise funds, the effect for couples is not significant. When we control for the herding effect in model 2, the gender effect persists, but its magnitude decreases to about 23%.

We control for platform effects in model 3. This specification includes categorical variables for each platform with CFP1 as the reference platform. Without the herding proxy, the gender effect for female entrepreneurs is still about 44%. There is no significant difference in the hazard of campaign completion between CFP1 and CFP2, whereas given the shorter campaign days cap on the CFP3 platform, the campaigns offered on the latter platform show a relatively slower hazard of almost 70%. In model 4 we add the herding proxy variable: this causes the female entrepreneur effect to decrease to about 20%, without altering the platform effects.

In model 5 and model 6 we interact the gender and the platform effects. All four interaction variables are insignificant. This provides evidence that the increased speed of campaign completion we find for female entrepreneurs may not be platform dependent. We perform a likelihood ratio test for nested models after estimation to compare the different model specifications [91]. The tests show that models 4 and 6 suit the data best, and given the insignificance of the four interaction terms of model 6, we use model 4 as our preferred specification.

The reported hazard ratios for the loan interest rates show that a 1% increase in the interest rate decreases the speed of campaign completion by 33%. This result seems rather intuitive if we take the interest rate to be a proxy for the riskiness of the project faced by the investor. When we control for the interest rate charged on the loans and the lower loan amount requested, we see that female entrepreneurs apparently do not “pay” for this increased speed of completion by accepting worse loan conditions. To verify this, we conduct an additional robustness analysis, where we interact the loan rate and the gender of the borrower, and find insignificant results. These findings are available upon request.

In line with other studies, providing a picture of the entrepreneur to the campaign information significantly increases the speed of campaign completion, indicating that prospective investors value the (soft) information they can infer from visual cues [12,50,54,55]. Campaigns on sustainable projects, however, consistently show a 34% decrease in the speed of completion. This finding is rather surprising, as Calic & Mosakowski [89] infer that sustainability oriented projects have a higher chance of funding in a reward based crowdfunding setting, and may be due to the relatively small number of sustainable campaigns present in our sample.

**Fig 2:**
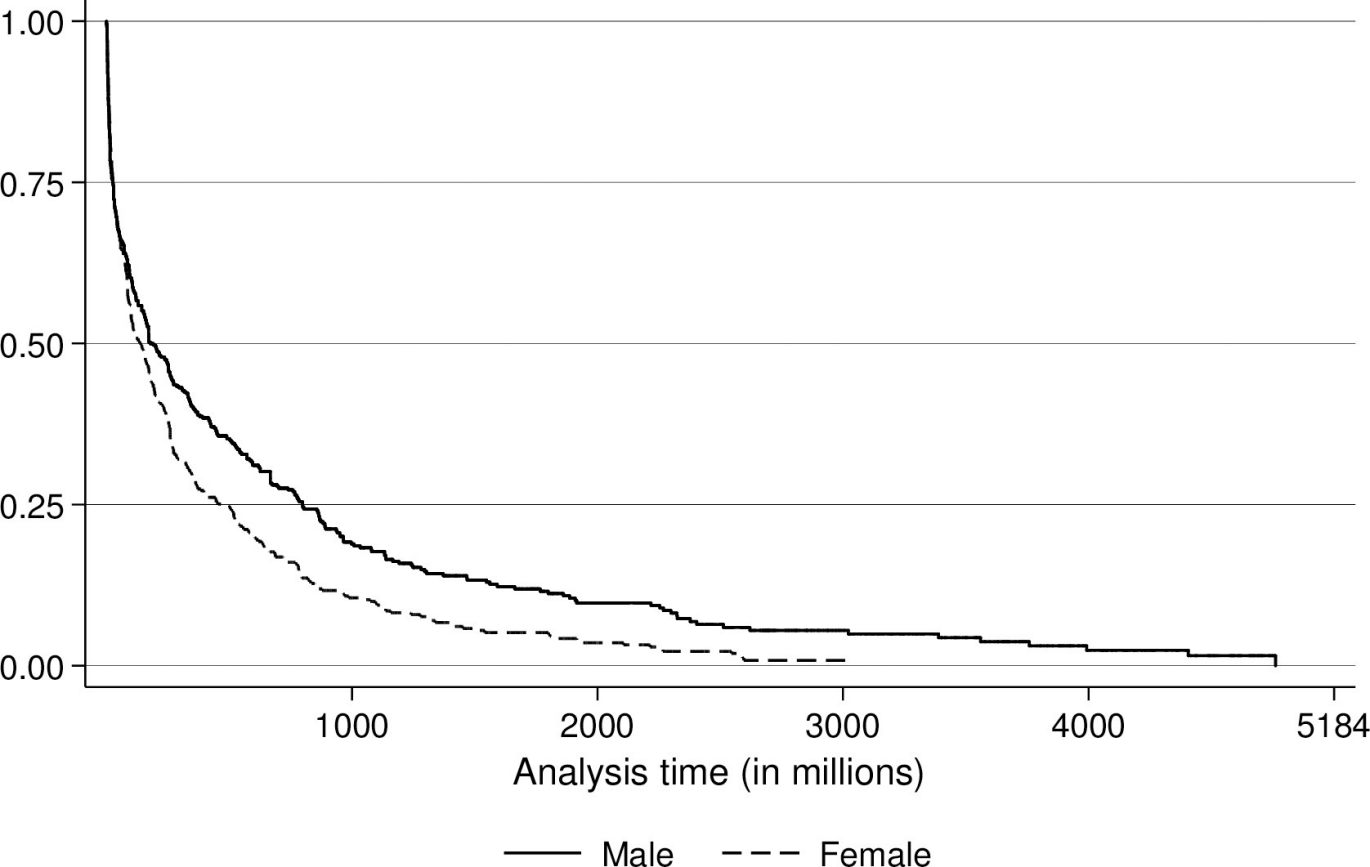
Kaplan-Meier survival curves by gender.

The Kaplan-Meier curve, or survival-function, shows the conditional probability of an individual surviving time t, given they reach time t [92,95]. The plot of the survival curves by gender is depicted in Fig 2. The figure shows how fast loan campaigns initiated by female entrepreneurs obtain the required loan amount compared to campaigns from male entrepreneurs. The horizontal axis is scaled in milliseconds, where 5,184,000,000 milliseconds correspond to 86,400 minutes, which is equivalent to 60 days. Recall that this is the maximum amount of campaign days allowed on CFP1 and CFP2. The log rank test for equality of survivor functions rejects the null hypothesis of no difference in survival between male and female entrepreneurs [91]. Hence, conditional on having a successful campaign outcome, female entrepreneurs have a shorter campaign completion time compared to male entrepreneurs.

### Survival analysis with shared frailty

In the current study, we benefit from campaign data stemming from three different Dutch crowdfunding platforms. As mentioned previously, these platforms are quite heterogenous in terms of the level of pre-screening of the entrepreneur and the business, and in terms of the (soft) information that is provided to prospective lenders. Measuring the, often qualitative, differences between platforms is impossible, but with the help of the frailty model we can still assess whether these differences affect our baseline results. In this section, we investigate whether certain entrepreneurs have shorter campaign completion times because of unobserved, platform-specific aspects, that may cause spurious duration dependency, and thus bias our estimates. In particular, we want to find out whether the role of female entrepreneurs in affecting campaign completion times changes once we take those unobserved, platform-specific aspects into account.

**Fig 3:**
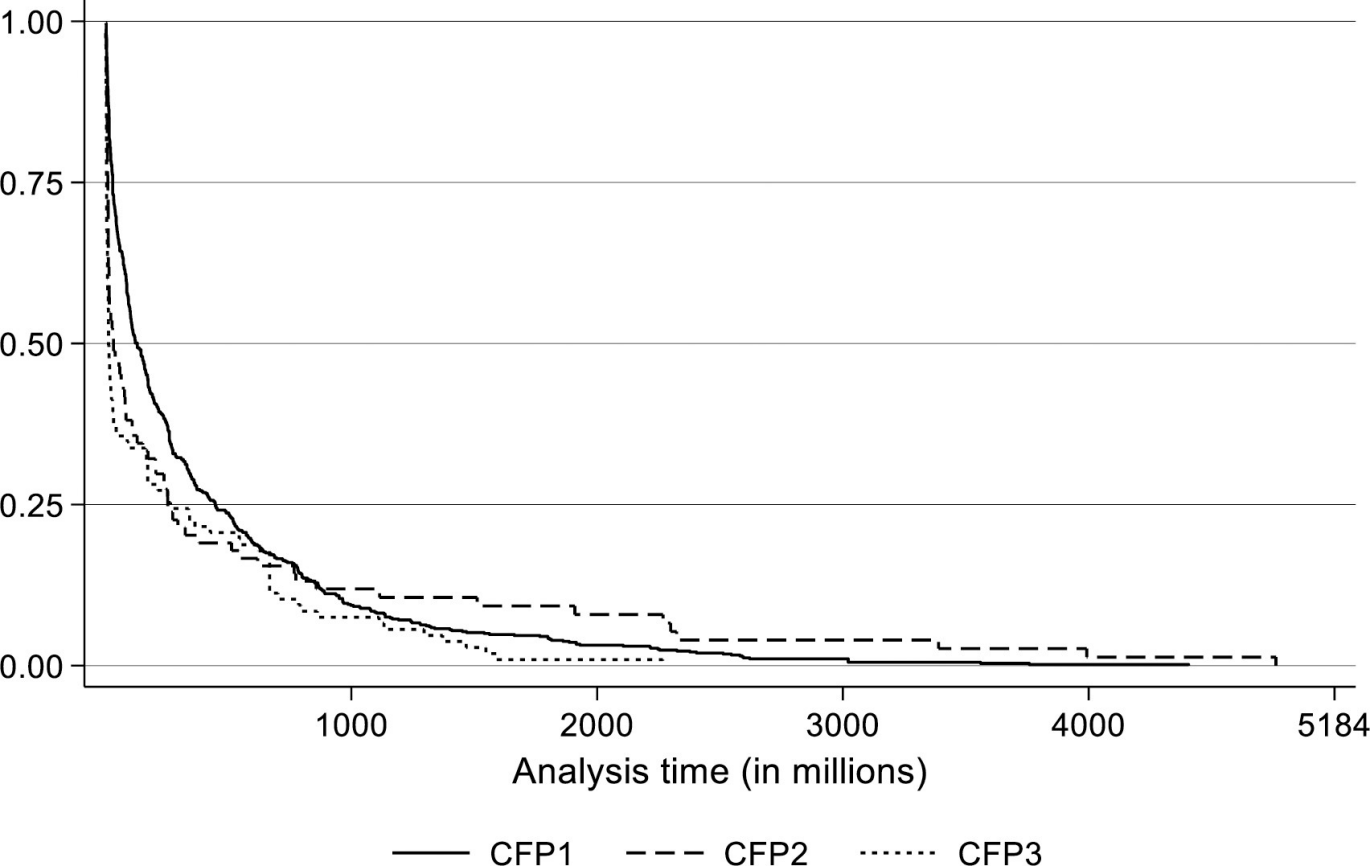
Kaplan-Meier survival curves by crowdfunding platform.

Fig 3 shows the Kaplan-Meier survival curves by platform. The curves show the survival probability as a function of the time it takes for an entrepreneur to obtain the required loan amount. For all three platforms, the survival patterns are very similar, and none of the platforms appears to significantly diverge from the others. Note the shorter campaign completion time for CFP3, which is due to the 31 days campaign time limit imposed by this platform.

In the presence of within-group correlation, crowdfunding campaigns offered on a given platform will share the same frailty. The estimated frailty variance is measured by *θ*. Table 4 reports the results of the Cox proportional hazards model with shared frailty. In the first column, model 1, we exclude our proxy variable for the herding effect. In the second column, model 2, we include the proxy variable. Both models show there is frailty variance; *θ* = 0.148 and *θ* = 0.153, respectively. Both variances are significantly different from zero, thus presenting evidence of the presence of within group correlation for campaigns listed on the same crowdfunding platform. Importantly for the purpose of our analysis, however, we find that this does not affect the role of female entrepreneurs much at all: accounting for intra-group correlation, we still find a positive and significant increase of about 20% in the hazard of campaign completion for female entrepreneurs, compared to male entrepreneurs.

**Table 4:** Time to crowdfunding loan campaign completion Cox proportional hazard rate model with frailty.

	(1)	(2)
VARIABLES	model 1	model 2
LN loan amount	2.2876***	1.3087***
	(0.0000)	(0.0000)
Loan maturity	1.0097***	1.0138***
	(0.0007)	(0.0000)
Loan interest rate	0.5692***	0.6576***
	(0.0000)	(0.0000)
Female entrepreneur (ref: male)	1.4439***	1.1979**
	(0.0000)	(0.0162)
Couples entrepreneur (ref: male)	1.2049	1.1371
	(0.1402)	(0.3094)
Picture entrepreneur	1.3994***	1.2615***
	(0.0001)	(0.0071)
Sustainable project	0.6329**	0.6640*
	(0.0421)	(0.0705)
Herding proxy, (Percentage obtained t-1)		1.0472***
		(0.0000)
Observations	14,365	14,365
Number of groups	3	3
Wald chi^2	666.4	1412
Theta	0.148	0.153
Std. Err. Theta	0.138	0.135
No. of campaigns	934	934
Funded end of sample	930	930

Robust p-values are reported in parentheses.

***, **, and * indicate statistical significance at the 1%, 5%, and 10% level, respectively.

### Robustness test for common support

According to Mollick [30], project maturity and the target amount of funds are negatively correlated with crowdfunding campaign success. In our data set, the campaigns initiated by female entrepreneurs are significantly shorter in maturity, and have lower requested loan amounts. To assess whether our results might be driven by the loan characteristics we split our sample between low and high loan amounts, and re-estimate our model on both sub-samples. The first approach is to split the sample at the median loan amount for the three types of entrepreneurs.

**Fig 4:**
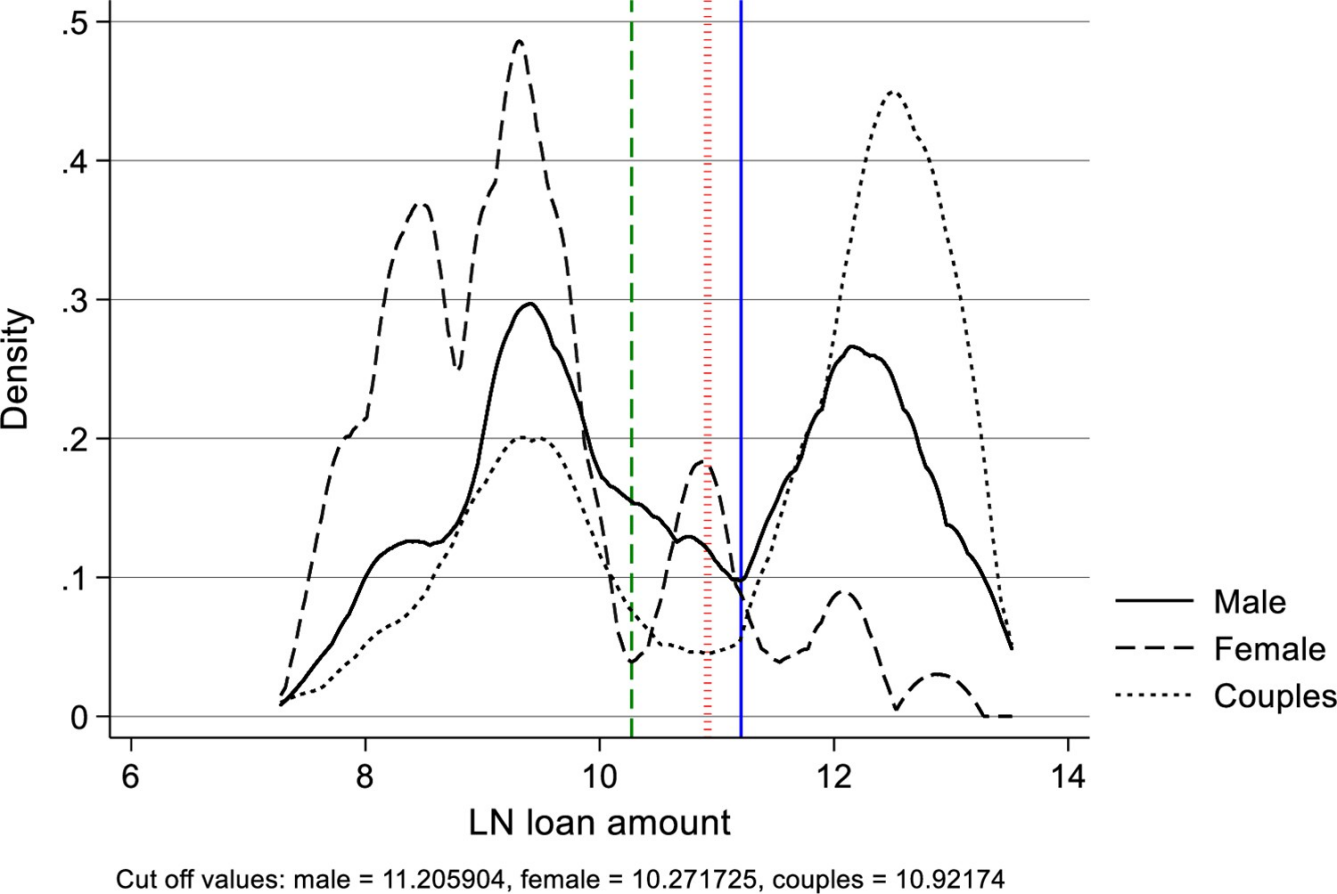
Kernel density plot for log loan amount. The kernel density plot shows the density distribution of the log loan amount value. The graph shows two peaks in the distribution for each entrepreneur type. We use the minimum value in the through between the peaks as cut-off point to split our sample.

The second approach is to plot the kernel density graph of the natural logarithm of the loan amount, and identify the appropriate cut-off for the common support. Fig 4 shows the kernel density plot by entrepreneur type. The kernel density estimation estimates the probability density function, where the area under the curve is equal to one, and the probability of the log loan amount being a certain value corresponds to the related area under the curve [91]. The vertical axis reports the density, and the horizontal axis reports the different values of the log loan amount. The plot shows two peaks for each entrepreneur type in the variable log loan amount. We split our sub sample for each entrepreneur at the minimum between the two peaks. This corresponds to values of 11.203 for male entrepreneurs, 10.272 for female entrepreneurs and 10.922 for couples. The colored vertical lines in the graph illustrate these cut off points.

Table 5 shows the results of both approaches. In our first approach, the median loan value for males is €13,200, for females it is € 4,600, and for couples € 26,200. These values are driven by the loans requested on the CFP1 platform, the largest share of observations in our dataset, and are well below the minimum loan values of the campaigns offered on both other crowdfunding platforms. This explains the results of columns (1) and (2) in the table. Nevertheless, the positive gender effect for female entrepreneurs persists. The second approach confirms our findings: splitting the sample with the help of our density graph shows that loans requested by female entrepreneurs have an increased hazard rate compared to male entrepreneurs, even more so when the requested loan amounts are relatively larger.

**Table 5:** Time to crowdfunding loan campaign completion, Cox proportional hazard rate model, split sample.

	Method 1		Method 2	
	(1)	(2)	(3)	(4)
	Below median	Above median	Left peak density	Right peak density
LN loan amount	3.8805***	1.4001***	1.6892***	1.7125***
	(0.0000)	(0.0000)	(0.0000)	(0.0000)
Herding proxy	1.0273***	1.0506***	1.0442***	1.0339***
(Percentage obtained t-1)	(0.0000)	(0.0000)	(0.0000)	(0.0000)
Loan maturity	1.0022	1.0099**	1.0227***	0.9916
	(0.7629)	(0.0500)	(0.0000)	(0.1772)
Loan interest rate	0.6515**	0.8255**	0.4787***	0.9241
	(0.0204)	(0.0302)	(0.0000)	(0.4312)
Female entrepreneur (ref: male)	2.4835***	1.7078***	1.2368**	1.7636***
	(0.0000)	(0.0002)	(0.0314)	(0.0052)
Couples entrepreneur (ref: male)	0.8404	0.9659	1.3530	1.0469
	(0.5206)	(0.8612)	(0.1382)	(0.8005)
Picture entrepreneur	2.0439***	0.9418	1.5516***	0.5087**
	(0.0000)	(0.6756)	(0.0001)	(0.0452)
Sustainable project	0.0000	0.8353	0.4684**	0.9490
	(.)	(0.2876)	(0.0159)	(0.8069)
CFP2	0.0000	0.5224*	3.9255***	1.0832
	(.)	(0.0821)	(0.0039)	(0.8639)
CFP3		0.2853***	4.2047*	1.2133
		(0.0018)	(0.0613)	(0.7226)
Observations	4,459	9,906	9,180	5,185
Wald chi^2	723.7	444.6	1149	146
No. of firms	475	459	731	203
Funded end of sample	475	455	730	200

Robust p-values are reported in parentheses.

***, **, and * indicate statistical significance at the 1%, 5% and 10% level, respectively.

### Cox proportional hazard rate by gender

An additional way to rule out possible platform effects for the increased hazard rate faced by female entrepreneurs is to repeat the survival analysis for each of the three entrepreneur types separately [95]. Table 6 shows the results of this approach. The variables CFP2 and CFP3, which capture the platform effects, are indeed insignificant for female entrepreneurs, confirming the earlier findings from our Cox proportional hazard model with frailty.

**Table 6:** Time to crowdfunding loan campaign completion, Cox proportional hazard rate model by gender of the entrepreneur.

	(1)	(2)	(3)
	Male	Female	Couples
LN loan amount	1.263***	1.515***	1.420**
	(0.000)	(0.000)	(0.013)
Herding proxy	1.048***	1.045***	1.046***
(Percentage obtained t-1)	(0.000)	(0.000)	(0.000)
Loan maturity	1.015***	1.017**	0.986
	(0.003)	(0.030)	(0.360)
Loan interest rate	0.751***	0.510***	0.908
	(0.001)	(0.001)	(0.691)
Picture entrepreneur	1.329**	1.298	1.036
	(0.031)	(0.102)	(0.925)
Sustainable project	0.676*	0.804	0.257**
	(0.069)	(0.604)	(0.034)
CFP2	0.632	1.779	0.310
	(0.209)	(0.419)	(0.293)
CFP3	0.247***	0.922	0.129**
	(0.000)	(0.913)	(0.035)
Observations	7,801	5,070	1,494
Wald chi^2	554.1	686.7	142.9
No. of campaigns	458	399	77
Funded end of sample	456	397	77

Robust p-values are reported in parentheses.

***, **, and * indicate statistical significance at the 1%, 5% and 10% level, respectively.

This analysis also reveals that the presence of a picture of the entrepreneur significantly increases the speed of campaign completion for males, but not for female entrepreneurs (although this effect is only marginally insignificant). Bearing in mind that providing a visual cue on the campaign platform is a non-random choice, it seems that our finding aligns with the studies by Baron et al. [60], Brooks et al. [61], and Patel & Wolfe [62]. The heterogeneity between the three platforms enables us to see that the gender effect for female entrepreneurs holds across campaigns that seek relatively smaller, but also relatively larger loans, and between campaigns initiated by Dutch and developing country female entrepreneurs.

### Alternative specification of the herding proxy

As some of the campaigns reach the requested loan amount in a very short time, the number of pledging moments we observe in the data can be limited. Therefore, we initially use a straightforward measure as our herding proxy: a lagged variable measuring the percentage of the loan amount reached in the previous scraping round. Another way to measure the herding effect is to create four categories of campaign completion: 0% to 25% of the loan amount funded, 26% to 50% of the loan amount funded, 51% to 75% of the loan amount funded and finally 76% to 100% funded, all in the previous scraping round, t-1. With the tranche from 0% to 25% of the loan amount funded serving as our reference category. This lagged herding variable then measures the *share* of the campaign that is completed in the previous period, instead of the *percentage* of the loan amount reached in the previous scraping round.

The estimation results are shown in Table 7. In line with the literature on herding behavior, we see in model 1 that the hazard of campaign completion is indeed greatest for the coefficient capturing the 76% to 100% share of the loan amount funded compared to the reference category. In model 2, we add the variables for the platforms and find results akin to the results in Table 3 with platform CFP3 showing a significant slower rate of completion than the other two platforms. Model 3 confirms the finding that there are no platform effects present regarding female and couples entrepreneurs. Finally, column 4 shows an interesting finding once we add interaction effects between the gender of the entrepreneur and the refined herding proxy. When adding these variables, the main effect for female entrepreneurs becomes insignificant, but the interaction terms for the three tranches of the loan amount are significant and show an increased speed of campaign completion compared to the reference category. This indicates that female entrepreneurs benefit from an increased speed of campaign completion compared to males, and that this gender effect may be driven by herding behavior in the loan crowdfunding campaigns present at these three platforms.

**Table 7:** Time to crowdfunding loan campaign completion, Cox proportional hazard rate model with alternative herding proxy.

	(1)	(2)	(3)	(4)
	model 1	model 2	model 3	model 4
LN loan amount	1.5337***	1.6251***	1.6264***	1.6198***
	(0.0000)	(0.0000)	(0.0000)	(0.0000)
Loan maturity	1.0089**	1.0082**	1.0076*	1.0078*
	(0.0257)	(0.0496)	(0.0721)	(0.0639)
Loan interest rate	0.5673***	0.6742***	0.6861***	0.6861***
	(0.0000)	(0.0000)	(0.0001)	(0.0001)
Female entrepreneur (ref: male)	1.2818***	1.2504***	1.2836***	0.8771
	(0.0023)	(0.0071)	(0.0065)	(0.3990)
Couples entrepreneur (ref: male)	1.0388	1.0365	1.4151*	1.7241**
	(0.8149)	(0.8340)	(0.0890)	(0.0279)
Picture entrepreneur	1.0363	1.3391***	1.3663***	1.3635***
	(0.6764)	(0.0077)	(0.0034)	(0.0035)
Sustainable project	0.5640***	0.5857***	0.5650***	0.5816***
	(0.0014)	(0.0072)	(0.0048)	(0.0072)
CFP2		0.6919	0.7364	0.7547
		(0.3226)	(0.4202)	(0.4597)
CFP3		0.2841***	0.2871***	0.2942***
		(0.0005)	(0.0008)	(0.0012)
26%-50% of loan amount funded t-1	1.0611	0.9951	0.9878	0.7162
	(0.6764)	(0.9724)	(0.9315)	(0.1150)
51%-75% of loan amount funded t-1	2.8127***	2.6855***	2.6725***	2.1418***
	(0.0000)	(0.0000)	(0.0000)	(0.0001)
76%-100% of loan amount funded t-1	12.6190***	12.0721***	12.0799***	10.1878***
	(0.0000)	(0.0000)	(0.0000)	(0.0000)
Female*CFP2			0.8672	0.8257
			(0.6362)	(0.5393)
Female*CFP3			1.0654	1.0048
			(0.8517)	(0.9893)
Couples*CFP2			0.5241	0.5749
			(0.2053)	(0.2608)
Couples*CFP3			0.5645*	0.6685
			(0.0902)	(0.3222)
Female* 26%-50% of loan amount funded t-1				1.9016**
				(0.0205)
Female* 51%-75% of loan amount funded t-1				1.7431**
				(0.0191)
Female* 76%-100% of loan amount funded t-1				1.5428**
				(0.0309)
Couples* 26%-50% of loan amount funded t-1				1.2710
				(0.6128)
Couples* 51%-75% of loan amount funded t-1				0.5000
				(0.1531)
Couples* 76%-100% of loan amount funded t-1				0.6979
				(0.3763)
Observations	14,365	14,365	14,365	14,365
Wald chi^2	1517	1469	1463	1510
No. of campaigns	934	934	934	934
Funded end of sample	930	930	930	930

Robust p-values are reported in parentheses.

***, **, and * indicate statistical significance at the 1%, 5% and 10% level, respectively.

## Conclusion

One of the main challenges for entrepreneurs is to acquire the necessary financial resources to fund their businesses. This challenge is even greater for female entrepreneurs as they possibly face discrimination when seeking funds through the more traditional lending channels like banks, who presently provide the lion’s share of financing for entrepreneurs [3,96]. Compared to their male counterparts, female entrepreneurs are less likely to obtain a bank loan, face worse credit conditions, and are required to pledge higher amounts of collateral, further increasing the hurdle to seek outside financing for their business [7–11,97].

One avenue to overcome the gender bias present in traditional lending channels is to make use of new financing instruments, like crowdfunding. Current research shows that in a crowdfunding setting, women have a higher likelihood to obtain funds, pay on average lower interest rates [12], and enjoy higher rates of campaign completion than men [21,23]. In essence, if more high-quality female borrowers are rejected in traditional loan markets because of the presence of a gender bias, these females will search for alternatives, and flow to crowdfunding markets instead. On average, these females are then more creditworthy than their male counterparts, and can signal this thanks to the greater amount of soft information they can provide on the crowdfunding platforms. Under this scenario, the “wisdom of the crowd” is better at assessing the borrower’s level of creditworthiness than individual loan officers are.

We contribute to the existing body of research on crowdfunding by investigating whether female entrepreneurs benefit from shorter campaign completion times compared to their male counterparts when requesting a business loan. Using data from three large Dutch crowdfunding platforms, we apply a Cox proportional hazard rate model and find that female entrepreneurs are not disadvantaged when they attempt to obtain a business loan through a crowdfunding platform. Instead, they benefit from a 20% shorter campaign completion time compared to campaigns initiated by male entrepreneurs. This effect is not present when couples request the loan.

One possible explanation for this shorter campaign completion time is that investors tend to put even more weight on the investment decisions of their peers when it comes to investing in loans requested by female entrepreneurs, and therefore tend to herd more. As said, investors gather information on the creditworthiness of the borrower by observing the lending behavior of their peers [16], and take the level of previous funding as a positive signal for their own investment decision [29]. In a crowdfunding setting, entrepreneurs have the opportunity to provide as much (soft)information as they desire [32,82], which decreases the level of asymmetric information beyond what is commonly feasible in traditional lending markets. This will benefit female entrepreneurs relatively more than their male counterparts.

Another contribution of our study is the comparative analysis we conduct amongst three heterogeneous platforms. The platforms in our dataset differ in various ways: in terms of the type of business loans they make available, the level of pre-screening of the entrepreneur and the business, the incremental amount of pledges, and the costs to the investors. Regardless of these differences between platforms, our findings are robust to a frailty approach, and show that female entrepreneurs have an advantage over their male counterparts in the hazard of campaign completion.

The implications of our findings are twofold. First, whilst policy makers increasingly recognize that getting women to start, and scale up their business is key to economic growth [6,98], access to finance is recognized to be a major hurdle. We provide evidence that alternative sources of financing such as loan-based crowdfunding render the necessary business funding more accessible to female entrepreneurs. The barriers present in traditional lending markets are overcome by the “wisdom of the crowd”: a large number of small investors demonstrates a greater ability to infer the creditworthiness of the entrepreneur by observing the lending behavior of their peers [16,76].

Second, we complement the existing entrepreneurial finance literature by showing that female entrepreneurs are not at a disadvantage compared to their male counterparts when it comes to business loans requested through crowdfunding. Previous studies have shown that women benefit in a pre-sale crowdfunding setting [22,23], and have revealed mixed results in loan settings for private purposes [12,13]. Interestingly, as we control for the interest rate charged on the business loans, and the lower loan amounts requested, female entrepreneurs apparently do not “pay” for the increased speed of campaign completion in loan-based crowdfunding by accepting worse loan conditions.

Our study comes with several limitations. From the available data we cannot infer whether it is taste-based, or statistical-based discrimination [12], that drives the investment decision of the lenders. We do not know whether the loan conditions offered by the entrepreneur match the business risk for the investor. Nor do we evaluate whether investors perceive the attributes of the entrepreneur to be aligned with their gender role, as would be predicted by role congruity theory. Related, we are unable to identify whether male or female investors provided the funding to the female entrepreneurs.

Regarding the investors, we cannot account for any soft information that they collect through the pictures, or videos, presented on the platforms. Investors are known to rely on visual cues offered by the entrepreneur, particularly in a context characterized by high levels of asymmetric information such as crowdfunding. Finally, it would be valuable to determine if the entrepreneur who requested a business loan through a crowdfunding platform also applied for a (similar) loan in a traditional lending market. Ideally, this information would be complemented with the knowledge of whether he/she was turned down by the lending institution, revealing if the request for a crowdfunding loan was a first or last resort type of decision. With that knowledge, we could further add to the stream of literature that aims to establish whether crowdfunding and bank lending are substitutes, or complements [99–101]. These questions constitute possible interesting avenues for future research.

## Supporting information

S1 Appendix Comparison table for the three crowdfunding platforms. (DOCX)
