# Supplementary material for: Gender effects in crowdfunded business loan campaigns
Source: PLoS One. 2024 Jul 10;19(7):e0305601. doi: 10.1371/journal.pone.0305601 (PMC11236130; doi:10.1371/journal.pone.0305601)
Supplement: S1 Appendix — (DOCX) [file pone.0305601.s001.docx]

**Table A. Comparison table for the three crowdfunding platforms.**

|  | CFP1 | CFP2 | CFP3 |
| --- | --- | --- | --- |
| Year of first campaign | 2013 | 2011 | 2014 |
| # campaigns to date | 2154 | 1374 | 557 |
| Total amount invested to date | €27 million | €136 million | €122 million |
| Assessment of loan quality | No credit rating | Credit rating | Credit rating |
| Determinant of loan quality | By local partners in foreign countries | By borrowers with minor advice from platform | By platform with extensive due diligence |
| Loan maturity | 6 to 48 months | 6 months to 10 years | 6 months to 10 years |
| Loan rate | 3%-6% | 4%-12% | 5%-9% |
| Costs to borrower | 3.10% | Publication fee and success fee | Credit evaluation costs, publication fee, administration fee and success fee |
| Minimum pledge | € 50 | € 100 | € 500 |
| Costs to investor | Free (with small credit card fee) | Success fee | Monthly administration fee |
| Campaign time | max. 60 days | max. 60 days | 31 days with possible extension |

Note.- This table compares the three crowdfunding platforms from which we retrieved the loan campaign information, and shows the relative differences among them for various characteristics.
